# Cases of Hysteria

**Published:** 1857-05

**Authors:** 


### ECLECTIC DEPARTMENT,

### AND SPIRIT OF THE MEDICAL PERIODICAL PRESS.

---

*Researches on the Cause of the Fluidity of the Blood.*—Dr. B. W. Richardson, in a paper read before the Chemical Section of the British Association for the Advancement of Science, commenced by giving a historical sketch of the various hypotheses which had been formed to account for the fluidity of the blood and the phenomena of coagulation. He then related his own investigations, which had led him to the discovery that ammonia is a constituent of the blood, and that on its presence the solubility of fibrin, and therefore the fluidity of the blood is dependent. The numerous experiments performed by the author were described: they may be thus classified: 1. By causing the vapor arising from coagulating blood to pass through another quantity of blood, drawn as nearly as possible at the same time and from the same animal, the coagulation of the latter is suspended so long as the current of vapor is kept up. 2. By driving the vapor of coagulating blood into pure hydrochloric acid, and afterwards treating with chloride of platinum, the characteristic yellow crystals of ammonio-chloride of platinum rea procurable. 3. On collecting a large quantity of freshly drawn blood in a wide-mouthed jar, and placing over it a cover, to the interior of which is fixed a slip of glass moistened with hydrochloric acid, the glass becomes covered with microscopic crystals of chloride of ammonium. 4. If fibrin removed from blood be carefully dissolved in a weak solution of ammonia, and again added to the serum and red particles, coagulation may be induced. The result arrived at was, that the phenomenon of coagulation depends essentially on the evolution of ammonia from the blood; and this gives an explanation of the modifications observed in the process of coagulation under the various physical conditions. In concluding his paper Dr. Richardson pointed out that ammonia, in combination with carbonic acid gas, is a constant constituent of the air expired in the breath. The presence of ammonia in the animal economy, and its evolution in respiration, was of interest in that it connected more closely the link that exists between the animal and vegetable worlds. But the subject was of the greatest importance in relation to the causes, the nature and the treatment of various diseases.—*Proceedings of British Ass. for Advancement of Science*, 1856, and *Amer. Jour. of Med. Science.*

*Cases of Hysteria.* By Wm. Wood, M. D,. of East Windsor Hill, Conn.

Case I.—*September* 24, 1851. I was called in great haste to see a Mr. L., aged about 55, some three miles distant. The messenger informed me that he was "sneezing himself to death," and he thought that he would be dead before I could get there. In fifteen minutes I was by his side, and found that the neighbors had already procured another physician, with his pupil, besides a housefull of friends. Dr. —— informed me that, on his arrival, Mr. L. was breathless and pulseless, and that his pupil had kept up artificial respiration several minutes before pulsation returned. As I entered the door, the anxious wife exclaimed, 'Oh, Doctor! can you save my husband? he is now just a-going to have another turn?" From what I gathered from the messenger, and what I saw on my arrival, I determined to administer immediately large doses of anti-spasmodics. I accordingly gave him at one dose

℞ Tinct. valerian. ammoniate ℥j;

Tinct. camphoræ gtt. xx;

Tinct. opii gtt. xl.

The effect was very marked, the paroxysms being entirely arrested. He remained quiet and easy about one hour, when, upon a return of the former symptoms, I again administered the above prescription with similar effect. I had occasion to give it but once more before the patient was entirely free from any trouble, except headache and some thirst, which continued for a few days.

Not knowing anything of the previous history of my patient, I catechized him particularly regarding his health, occupation, habits, &c. &c., by which I learned that he had been an engraver for several years in Albany, but of late had given up the business, and bought a small farm, as advised by his physician, under the belief that applying himself so closely to his trade was injuring his health. While residing in Albany, he was afflicted with a slight bronchial difficulty, but had experienced no trouble from it since leaving the city, unless he went near salt water, when it caused some hoarseness. His health is tolerably good; eats well; sleeps well; and generally feels well. He never had anything that bore the least resemblance to this attack before. The day on which this occurred, Mr. L. felt as well as usual, and was engaged in some light work about his farm. Toward evening, he went to his barn to milk, and while milking, suddenly commenced sneezing, and could not stop. He started to go into the house, but was obliged to leave his milk pail, and catch hold one of the studs of the barn to keep from falling, and was found in this condition by his hired man, who helped him into the house; this is the last he remembers until some time after I arrived. The sneezing continued until after he was breathless, accompanied by severe spasms.

*Nov.* 19, 1856. A little more than five years after the first attack, I was summoned in great haste to see Mr. L. again, with the message that he was 'coughing himself to death.' I was there as soon as possible, but found him coughing constantly, and in so violent convulsions, that I was unable to administer anything, either by mouth or rectum. It took three men to keep him on the bed, and to prevent him injuring himself by his spasmodic movements. He soon became very purple, rigid and breathless. I placed my ear over his heart, but found all was silent there. The muscles all over him were as tense as fiddle strings, and his jaws were firmly closed. I then resorted to Dr. Marshall Hall's method for the treatment of asphyxia, so far as practicable, as laid down in the No. of the *American Journal of the Medical Sciences* for July last. The rigidity soon passed away, and he expressed himself as feeling comfortable, with the exception of very great soreness externally. He remained quiet about one hour, and then commenced coughing again. I immediately gave him the same dose as in his previous attack. It did not fully arrest the spasms, but made them comparatively light. There were two more returns of coughing, but the paroxysms were entirely checked by increasing the dose about one-third.

The next day I found him with pulse 90; tongue coated white; pain on touching him very acute, and with a congested appearance about the neck and face, and considerable fullness of the face. He was perfectly rational, and seemed desirous to tell me just how he felt before the paroxysm. He said, 'Doctor, I never felt better in my life than I did before I commenced coughing. I had been sitting in the house most of the day, reading and singing some of the time, but about supper time I began to feel a little bad, and I was afraid that I was going to have a poor spell. I made up my mind that I would keep as composed as possible, and try to remember all my feelings, so as to tell you. I first felt a prickling sensation in my left side, in the region of my kidney, similar to what I feel in my fingers when I come to the fire after being in the cold. After a few minutes it seemed to extend up my ribs, until it reached my lungs, when it produced a sensation there, similar to what I feel when I strike my elbow against a hard substance, and hit the nerve. I then began to cough, and in spite of all my efforts to the contrary, I kept coughing, and could not get time to take in a breath; it was all expiration, and this continued until I became unconscious.'

This I suppose to be a case of hysteria, and if so, it is peculiar in several respects. 1st. Hysteria is uncommon in men. Gregory says; 'Hysteria is scarcely ever observed except in females.' 'Cases of hysteria have been recorded in *males*, but upon no very good authority; the complaint is peculiar to the female sex." Mackintosh says: 'It is a disease almost exclusively affecting females, but males are not entirely exempt, but the attacks come on all of them under the influence of *depressing passions*." 2. Hysteria is uncommon at Mr. L.'s period of life. Watson says: 'Etymologically to apply the term hysteria to males would be absurd, but it does present itself, though, rarely, in *young men*." 3d. Hysterical sneezing very rarely occurs, and I am unable to find an instance of it in the male. R. B. Todd, Phys. to King's Col. Hosp., says: '*Women* are sometimes attacked with hysterical fits of sneezing; of my own knowledge, I am aware of but one instance of this.' Sir Benjamin Brodie in his extensive practice relates only two cases of this kind, and they occurred in *females*.

There was in this case no *exciting or depressing* cause that I could learn, neither could I trace the proximate cause of his attacks to any disturbance.

Case II.—*August* 24, 1856. I was summoned in great haste to see two sisters, one aged 20 and the other 18, who were very suddenly and alarmingly taken sick. Before reaching the house I could hear them laboring for breath. I found them in violent convulsions, with great rigidity of the muscles. In taking hold of their arms, I could compare the rigidity to nothing but to that of a bar of iron. Their breathing was short, hurried and loud; about 130 expirations to the minute; pulse 80 to 90; constant retching. One vomited very freely, the other not at all, and it is a singular fact that from a child it had been impossible to make her vomit. The first words that each of them spoke, were, 'rub me, rub me,' although they were in different rooms. As soon as practicable I gave each of them a pill of the following:

℞ Sulph. morph. gr. iv;

Gum camp. ℈ij;

Ex. conii. q. s.—Ft. pil. xii.

One of these pills were given whenever there was an approach of a paroxysm, and in the meanwhile I directed them to take mucilaginous drinks very freely. They made a good recovery, but complained of excessive soreness for several days.

The youngest was affected the last, and after the abatement of the spasms, related the following: 'Both of us went to meeting to-day, and felt as well as usual; drank tea about 7 o'clock—good appetite—eat biscuit, cake, new cheese, and whortleberry pie. At about half-past seven I heard my sister make a noise in the adjoining room, and started to go to her, but before reaching the door my limbs began to tremble, and felt so stiff that I was unable to move them. I should have fallen had I not been caught by a friend who was by my side. I thought I was dying, and attempted to speak, but could not, for it seemed to me as though a girth was buckled so tight across my chest that I could neither speak nor breathe. Then commenced the loud and difficult breathing, accompanied with spasms, nausea, head-ache, thirst, and great burning in my stomach.'

The older sister had been taken a few minutes before with nausea and vomiting, and then followed the symptoms as described above, only much more violent. She described the heat in h r stomach as a 'ball of fire burning through.' They both described the sensations when the convulsions were passing off, as the most distressing of all, when they cried out, 'rub me, rub me.' They said 'they felt *all over* like a limb that had been asleep and was waking up.'

My first impression on seeing them was that it was hysteria, but upon hearing the whole story, it appeared as though tney must have taken some violent poison. Although Sydenham has truly observed, 'that the shapes of Proteus, or the colors of the chameleon are not more numerous and changeable than the variation of hysteric complaints,' yet it seemed to me impossible for hysteria to assume the whole category of symptoms attendant upon poison by nux vomica. The manner of attacks, both taken at the same time without seeing each other, inability to move, the regular natural pulse, the great rigidity of the muscles, the burning heat in the stomach, the constriction of the chest, the pricking and tingling sensation all over, are symptoms produced by nux vomica; the other symptoms ordinarily occur in hysteria, as well as violent poisoning. I questioned them to know if they could have taken anything poisonous, but they were positive that they had not. I have no doubt but what they told the truth, for both of them are excellent young ladies, members of the church, and are very pleasantly situated to enjoy life. I then examined the pie, but could find no poisonous berries in it, and I knew of no berry in this section that is so violent a poison. Could it have been the cheese? Cheese is as violent and fatal a poison to some as nux vomica. They have, however, always eaten cheese with impunity, and furthermore, there were eleven at the table, and all eat of the cheese and pie, and only two were taken sick, and they were as robust and healthy as any member of the family, and more so, for every member except these two had been sick with dysentery within two months previous. What caused the attack?

[This second case appears to us to have been one of hysteria brought on by indigestion—*embarras gastrique.*—Ed.]—*American Journal of Medical Sciences.*

